# Editorial: Neuropsychiatric consequences of the COVID-19 pandemic: understanding mechanisms, risk factors, and treatment

**DOI:** 10.3389/fnhum.2025.1611176

**Published:** 2025-05-08

**Authors:** Zulay R. Lugo, Mario Jose Patiño-Torres

**Affiliations:** ^1^Department of Neurology, University Hospital of Caracas, Central University of Venezuela, Caracas, Venezuela; ^2^Department of Internal Medicine, University Hospital of Caracas, Central University of Venezuela, Caracas, Venezuela

**Keywords:** COVID-19, sleep, long COVID, mental health, neuropsychiatric (NPS) complications

The COVID-19 pandemic caused by the novel coronavirus SARS-CoV-2, began in late 2019 and its neurological and psychiatric manifestations have become evident. Already during the acute phase of the disease neurological abnormalities were detected, including decreased sense of smell and taste, peripheral neuropathies such as Guillain-Barré syndrome, encephalitis, stroke, and seizures (Taher et al., 2021).

Psychiatric manifestations associated with the development of COVID-19 were also found at the acute phase of the disease. A meta-analysis showed a higher prevalence of depression and post-traumatic stress disorder in affected patients (Vindegaard and Benros, 2020). Cases of psychosis, anxiety, manic episodes, and depressive episodes with psychotic symptoms, triggered or appearing in the context of SARS-CoV-2 infection, were also reported (Forero-Peña et al., 2022).

Besides the direct symptoms caused by the coronavirus, the mandatory quarantine period imposed in most countries led to the appearance or worsening of psychiatric symptoms, such as depression and anxiety, and the occurrence of new symptoms, such as sleep disorders and post-traumatic stress disorder. These symptoms have been described both in patients (Pappa et al., 2022) and in the general non-infected population (Casagrande et al., 2020). Events related to the confinement period, for example, excessive alcohol intake and experiences of physical and emotional abuse, have been reported (Morales et al., 2021). Finally, late manifestations of the disease, including so-called long COVID, may include neurological and psychiatric symptoms such as headache, attention disorders, and cognitive dysfunction (Rodriguez-Morales et al., 2023).

This Research Topic aimed to collect reports on these manifestations, as well as their possible pathophysiology and management: they cover (1) neurological or psychiatric manifestations during the symptomatic period of the disease, (2) psychological, psychiatric, and social manifestations as a direct consequence of the quarantine period, and (3) neurological or psychiatric manifestations developed after the resolution of the acute phase of the infection which are included in the so-called post-COVID syndrome.

In this context, two of the main symptoms of the SARS-CoV-2 infection were alterations in smell and taste. A recent review found that anosmia was the most common symptom among these disorders (Mehraeen et al., 2021). In some cases, acute olfactory dysfunction might have evolved into chronicity. The review article presented in this Research Topic by Treder-Rochna et al. showed that this complication may be effectively treated with olfactory training and other adjuvant therapies.

Among the psychological aspects of the pandemic touching those who did not directly suffer from the disease, the vast amount of information on the SARS-CoV-2 infection disseminated through the media may have produced a shift in attention in some people due to the possible development of a state of generalized anxiety, affecting the detection of specific negative stimuli. This perceptual change is shown with the work of Favieri et al. presented in this Research Topic. These findings highlight the extent of the possible impact on mental health during the pandemic, not directly related to infection by SARS-CoV-2.

In this Research Topic, Liu et al. present a study about the effects on sleep and mental health in a population of patients with SARS-CoV-2 infection, assessed through a questionnaire distributed via a social media platform. Among these patients, they found lower sleep quality, longer sleep latency, enhanced rising time, and decreased sleep efficiency after the wave of infection. These alterations were significantly correlated with anxiety and depression, among other psychological factors.

Finally, long COVID, as defined by WHO, occurs in individuals with a history of probable or confirmed SARS-CoV-2 infection, usually 3 months from the onset, with symptoms that last for at least 2 months and cannot be explained by an alternative diagnosis (Soriano et al., 2022). It can include symptoms from a variety of systems, such as the respiratory and cardiac systems, in addition to central nervous system symptoms, such as dizziness, brain fog, attention disorders, and cognitive dysfunction (Rodriguez-Morales et al., 2023; Thaweethai et al., 2023). The diversity of manifestations of this syndrome means that its diagnosis and therapeutic approaches are still subject to debate and further research. On this subject, Smadja and Abreu propose in this Research Topic a preliminary therapeutic approach for long COVID based on whole body hyperthermia (WBH), which would act through the modulation of neuroinflammation. This therapy could also have potential in the treatment of some neurodegenerative diseases such as Parkinson's disease, Alzheimer's disease, Huntington's Disease, and various spinocerebellar ataxias, which share a pathology characterized by the accumulation and deposition of abnormal polypeptides or amyloid products and might be modulated by WBH, according to these authors.

The articles presented in this Research Topic provide an excellent example of the diverse range of neuropsychiatric symptoms and pathologies associated with COVID-19, along with some therapeutic approaches. We hope that this contribution will help to increase the body of scientific knowledge on this field, especially on the long-term COVID neuropsychiatric symptoms and their possible management, as well as to encourage further research on this Research Topic.
